# Spatial Risk Effects From Lions Compound Impacts of Prey Depletion on African Wild Dogs

**DOI:** 10.1002/ece3.70401

**Published:** 2024-10-18

**Authors:** Ben Goodheart, Scott Creel, Paul Schuette, Egil Droge, Justine A. Becker, Kambwiri Banda, Anna Kusler, Stephi Matsushima, Kachama Banda, Ruth Kabwe, Will Donald, Johnathan Reyes de Merkle, Adrian Kaluka, Clive Chifunte, Matthew S. Becker

**Affiliations:** ^1^ Department of Ecology Montana State University Bozeman Montana USA; ^2^ Zambian Carnivore Programme Mfuwe Eastern Province Zambia; ^3^ U.S. Fish and Wildlife Service Marine Mammals Management Anchorage Alaska USA; ^4^ Wildlife Conservation Research Unit, Department of Zoology, The Recanati‐Kaplan Centre University of Oxford Tubney UK; ^5^ Musekese Conservation Kafue National Park Lusaka Province Zambia; ^6^ Zambia Department of National Parks and Wildlife Chunga Central Province Zambia

**Keywords:** intraguild competition, *Lycaon pictus*, prey depletion, space‐use, subordinate carnivore

## Abstract

Prey depletion threatens many carnivore species across the world and can especially threaten low‐density subordinate competitors, particularly if subordinates are limited to low densities by their dominant competitors. Understanding the mechanisms that drive responses of carnivore density to prey depletion is not only crucial for conservation but also elucidates the balance between top‐down and bottom‐up limitations within the large carnivore guild. To avoid predation, competitively subordinate African wild dogs typically avoid their dominant competitors (lions) and the prey rich areas they are associated with, but no prior research has tested whether this pattern persists in ecosystems with anthropogenically‐reduced prey density, and reduced lion density as a result. We used spatial data from wild dogs and lions in the prey‐depleted Greater Kafue Ecosystem to test if wild dogs continue to avoid lions (despite their low density), and consequently avoid habitats with higher densities of their dominant prey species. We found that although lion density is 3X lower than comparable ecosystems, wild dogs continue to strongly avoid lions, and consequently avoid habitats associated with their two most important prey species. Although the density of lions in the GKE is low due to prey depletion, their competitive effects on wild dogs remain strong. These effects are likely compounded by prey‐base homogenization, as lions in the GKE now rely heavily on the same prey preferred by wild dogs. These results suggest that a reduction in lion density does not necessarily reduce competition, and helps explain why wild dogs decline in parallel with their dominant competitors in ecosystems suffering from anthropogenic prey depletion. Protecting prey populations within the few remaining strongholds for wild dogs is vitally important to avoid substantial population declines. Globally, understanding the impacts of prey depletion on carnivore guild dynamics should be an increasingly important area of focus for conservation.

## Graphical abstract

Spatial avoidance of lions by wild dogs has never been tested in a prey and lion‐depleted ecosystem. We found that wild dogs continue to avoid lions and prey even though both have been reduced. Mechanistically, this helps to explain the lack of competitive release of wild dog populations when lions are reduced as a result of prey depletion.

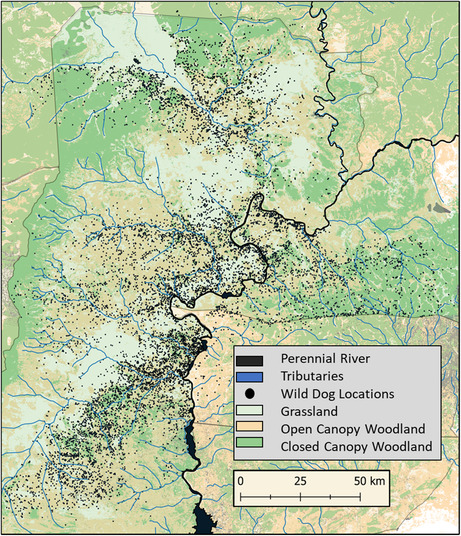

## 1 Introduction

Large carnivores have disproportionate impacts on their ecosystems, but are among the world's most threatened taxa (Carbone and Gittleman 2002). Most large carnivore species now occur over just a small fraction of their former range, and the majority of carnivore populations are declining rapidly, particularly in areas experiencing high rates of human population growth, such as sub‐Saharan Africa (Ripple et al. 2014). Large carnivores are conflict‐prone and require large contiguous areas with adequate prey populations to meet their energetic needs, and this makes conservation of free roaming populations difficult. In Africa, the majority of viable large carnivore populations, particularly African wild dogs (*Lycaon pictus*, wild dogs hereafter) and lions (*Panthera leo*) occur in protected areas (Riggio et al. 2013; Bauer et al. 2015; Woodroffe and Sillero‐Zubiri 2020; Bodasing 2022) that are increasingly isolated due to habitat encroachment and loss (Brooks et al. 2002; Lindsey et al. 2014; Watson et al. 2015; Wolf and Ripple 2017). Remaining protected areas that are large enough to sustain healthy large carnivore populations, are increasingly threatened by depletion of their large herbivore prey (Craigie et al. 2010; Lindsey et al. 2013, 2017; Ripple et al. 2015). Prey depletion is an emerging threat in many parts of the world and can have strong and cascading impacts on ecosystem function (Estes et al. 2011). Negative effects of prey depletion on the conservation of large carnivore populations in protected areas are beginning to emerge, and vary appreciably among species (Datta, Anand, and Naniwadekar 2008; Steinmetz, Seuaturien, and Chutipong 2013; Wolf and Ripple 2016; Carter, Levin, and Grimm 2019; Goodheart et al. 2021; Vinks, Creel, Rosenblatt et al. 2021; Vinks, Creel, Schuette et al. 2021; Creel et al. 2023).

The African wild dog is an endangered carnivore with an estimated 6600 individuals left in the wild (Woodroffe and Sillero‐Zubiri 2020). Wild dogs are limited by human threats including habitat loss, disease, direct persecution (Fanshawe, Frame, and Ginsberg 1991; Alexander and Appel 1994; Kat et al. 1996; Woodroffe and Ginsberg 1999; Creel and Creel 2002; Woodroffe et al. 2007; Prager et al. 2011), and by their dominant competitors, African lions and spotted hyena, (*Crocuta crocuta*, *hyena hereafter*) (Fanshawe and Fitzgibbon 1993; Mills and Biggs 1993; Creel and Creel 1996; Mills and Gorman 1997; Gorman et al. 1998; Broekhuis et al. 2013; Swanson et al. 2014; Speakman et al. 2015; Groom, Lannas, and Jackson 2016). Both lion and hyena densities are tightly correlated to the density of large herbivore prey (Orsdol, Hanby, and Bygott 1985; Hayward, O'Brien, and Kerley 2007; Hatton et al. 2015), but wild dog density does not correlate with prey density in the same manner. Instead, wild dog densities have an inverse relationship with dominant competitor densities, so that wild dog populations decline as lion and hyena populations increase (Creel and Creel 1996, 2002; Mills and Gorman 1997; Vucetich and Creel 1999; Groom, Lannas, and Jackson 2016). Prey depletion reduces lion densities within ecosystems (Lindsey et al. 2017; Vinks, Creel, Schuette et al. 2021), and it would be logical to assume that wild dogs experience competitive release in response. Upon testing this expectation, we have previously found that wild dogs do not experience competitive release when their dominant competitors are reduced by prey depletion (Goodheart et al. 2021). Even with abundant prey, wild dog densities are consistently much lower than those of lions or spotted hyenas (Creel and Creel 1996, 1998, 2002; Creel, Mills, and McNutt 2004; Swanson et al. 2014; Groom, Lannas, and Jackson 2016; Goodheart et al. 2021); and because their density declines in parallel with their dominant counterparts when prey are depleted, wild dog populations are particularly at risk of reaching extinction thresholds in prey depleted systems (Goodheart et al. 2021).

The mechanisms that limit wild dogs in prey depleted systems are not well studied. Wild dogs operate on a tenuous energy budget that can be substantially impacted by competition with dominant competitors (Gorman et al. 1998). Prey depletion may exacerbate these effects not only because there are fewer resources on the landscape but also because competition for those remaining resources could remain strong as a result of prey‐base homogenization and increased dietary niche overlap within the large carnivore guild (Creel et al. 2018). Movement is among the most energetically costly activities that wild dogs undertake (like all terrestrial vertebrates), and wild dog movements are strongly influenced by lions even when lion density is low as a result of prey depletion (Goodheart et al. 2022). This suggests that competitive effects of lions on wild dogs might remain strong even if lion density is low, perhaps with energetic costs that scale up to the population level (Goodheart et al. 2022).

The use of space within a territory is based on movements aimed at maximizing foraging success, and minimizing the energetic costs associated with the acquisition and defense of resources, breeding opportunities, and avoiding predation within an individual's territory (MacArthur and Pianka 1966; Pyke 1979; Macdonald 1983). Spatial avoidance of lions by wild dogs is well documented (Creel and Creel 1996; Mills and Gorman 1997; Vanak et al. 2013; Darnell et al. 2014; Swanson et al. 2014; Groom, Lannas, and Jackson 2016; Dröge et al. 2017a), and wild dogs must balance trade‐offs between avoiding dominant competitors while maintaining access to prey, which can carry energetic costs because lions generally select areas with high prey densities. Prey depletion reduces the density of dominant competitors, and the combined effects on the space‐use of wild dogs in prey depleted systems have never been described. On one hand, low lion density could reduce the need for spatial avoidance by wild dogs, thus reducing costs of competition. On the other hand, low lion density may not reduce these costs, because competition for the remaining resources remains strong (Creel et al. 2018; Goodheart et al. 2022). Because prey depletion increasingly threatens many remaining wild dog populations, it is important to determine the effects of prey depletion on wild dog space‐use to inform conservation and management.

Using a resource utilization framework (Marzluff et al. 2004), we tested how wild dog space‐use was affected by lions, known environmental predictors of prey density, and anthropogenic variables in the Greater Kafue Ecosystem (GKE), which has low densities of wild dogs (0.79 individuals/100 km^2^; Goodheart et al. 2021) and lions (3.4 individuals/100 km^2^; Vinks, Creel, Schuette et al. 2021) as a result of anthropogenic prey depletion (Creel et al. 2018; Schuette et al. 2018; Vinks et al. 2020). Specifically, we created utilization distributions (but see also “occurrence distributions” – Alston et al. 2022) from dynamic Brownian bridge movement models (dBBMMs) fit to more than 13,000 locations from GPS‐collared wild dogs from 2018 to 2022. We then tested for covariates that influenced the distribution of wild dogs to reveal what processes affect wild dog space use in an ecosystem with anthropogenic prey depletion and niche compression within the large carnivore guild. We ran identical tests on utilization distributions of lions fit to more than 100,000 locations from 2018 to 2022, to compare habitat associations between dominant and subordinate competitors (lions and wild dogs) and relate them to habitat preferences for the dominant prey species of these two competitors in the GKE (Matandiko 2016; Creel et al. 2018; Schuette et al. 2018; Vinks et al. 2020).

## 2 Methods

### 2.1 Study Area

This study was conducted in the northern and central portions of the Kafue National Park (KNP) and the surrounding Game Management Areas (GMAs) of Kasonso‐Busanga and Namwala (located in central Zambia [S 14.5394, E 26.0782]). Kafue National Park is the second largest national park in Africa and totals 22,319 km^2^. The national park is strictly protected, but due to its massive size and the limited resources available to park managers, it has undergone substantial prey depletion (Midlane 2014; Overton et al. 2017; Vinks et al. 2020). GMAs surrounding KNP have communities living in them, and are managed for multiple uses including farming, fishing, trophy hunting, and wildlife protection. The national park and surrounding GMAs make up the 66,000 km^2^ GKE which forms the entire northern portion of the Kavango Zambezi Trans‐frontier Conservation Area (KAZA), which spans Angola, Botswana, Namibia, Zambia, and Zimbabwe. The core of the GKE is centered around the Kafue River and its tributaries and is a mosaic of woodlands dominated by miombo species (*Brachystegia* and *Julbernadia* spp.) interspersed with acacia woodland, riverine woodland, termitaria woodland, savannah grassland, and seasonally inundated grasslands. On average the region annually receives about 1020 mm of rainfall, with a rainy season characterized by extensive flooding that occurs between December and April and a dry season between May and November.

### 2.2 Data Collection

Using methods described in Goodheart et al. (2022), we deployed satellite GPS collars (Model TGW 4270: Telonics Inc., Mesa Arizona, USA) on at least one individual in 10 wild dog packs between 2018 and 2022 for a total of 34 pack‐years. We excluded single‐sexed groups of dispersers from our analysis and only focused our investigation on resident breeding packs. Wild dog locations were recorded twice daily at morning (08:00 or 08:30) and evening (18:00 or 19:00), typically following crepuscular activity periods, for a total of 17,392 locations. Because wild dogs move as a highly cohesive unit, we analyzed data from one individual in each pack (usually a breeding adult) and supplemented missing locations due to satellite connection failure using other collars in the pack when available. We also deployed satellite GPS collars (Model TGW‐4570, Telonics Inc., Mesa, Arizona, USA) on one adult female in 16 lion prides from 2018 to 2022 for a total of 41 pride‐years. Lion locations were recorded at 4‐h intervals for a total of 113,670 locations.

We immobilized both wild dogs and lions by intermuscular injection of medetomidine and tiletamine—zolazepam, reversing the medetomidine by intramuscular injection of atipamezole after 45 min to 1 h. Anesthetics were delivered by darting with an air‐powered DanInject rifle. All procedures were carried out by an experienced Zambian‐registered veterinarian in collaboration with the Zambia Department of National Parks and Wildlife, and with an approved protocol by the MSU IACUC (approval number 2020‐123). As in prior studies (Rosenblatt et al. 2014; Mweetwa et al. 2018; Goodheart et al. 2021; Vinks, Creel, Schuette et al. 2021; Creel et al. 2024), we tested for an effect of collaring on survival for both wild dogs and lions and confirmed that the mortality rate of radio‐collared individuals was not higher than the mortality of uncollared individuals.

### 2.3 Estimating Space‐Use

#### 2.3.1 Wild Dogs

To estimate wild dog space‐use in the GKE we created utilization distributions by fitting dynamic Brownian bridge movement models using the R package *move* (Kranstauber, Smolla, and Scharf 2020). We examined space‐use at annual and seasonal scales. To test for seasonal effects on space use, we partitioned the data into two annual periods, a rainy season which we defined as December 1st–April 30th and a dry season which we defined as May 1st–November 30th. To compare these seasonal patterns to space‐use across an entire year, we used three partitions of data (full year, dry season, wet season) for every year. For each partition of data we calculated dBBMM utilization distributions (UDs) for each pack using a window size of 15 locations and a margin size of five locations following guidance from Kranstauber et al. (2012). For more details on our use of dBBMMs with these data see Goodheart et al. (2022). UDs were calculated for each pack, rasterized at 1 km^2^ resolution, and summed to account for areas of overlap between wild dog packs to create a single layer measuring the intensity of wild dog use. In total, 15 of these summed utilization distributions were created, for the wet season, dry season and full year in each of 5 years.

#### 2.3.2 Lions

To create a variable quantifying the risk of encountering lions we employed the same method of fitting dBBMMs (just described) to our lion data, following methods from Goodheart et al. (2022). We used a window size of 35 locations and a margin size of 7 locations. We fit dBBMMs to seasonally and yearly partitioned data in the same fashion as the wild dog analysis above, to enable direct comparison of space‐use between wild dogs and lions for specific years and seasons. We rasterized utilization distributions for each pride within a given year and season, and summed them using the same 1 km^2^ grid as our wild dog UDs, to create a single layer measuring the intensity of lion space‐use. For each of these seasonal and annual UDs, we also created an alternative measure of lion space‐use, with UD values from the dBBMMs weighted by the associated pride's size (range 1–14 individuals) for the given year (i.e., multiplying raster cell UD values by the number of adults and subadults over 2 years of age in the pride). Again, we summed these weighted pride level UDs to account for areas of overlap and create a single layer measuring the intensity of lion space‐use. This created a lion UD layer in which areas occupied by larger prides have larger values than areas occupied by smaller prides, given the same probability of use. Pride sizes were calculated by direct observations from intensive monitoring during the duration of the study in which all pride members were known using methods described by Mweetwa et al. (2018) and Vinks, Creel, Schuette et al. (2021). Lion prides undergo fission‐fusion dynamics, but lions in the GKE form small prides (Vinks, Creel, Schuette et al. 2021), which have lower rates of fission‐fusion (Mbizah et al. 2019). In addition, most lion prides in our study have been collared over multiple generations and have shown little variation in ranging patterns by different collared individuals. For both wild dog and lion dBBMMs we used an average location error of 2 m based on previous work showing a mean location error of 1.89 m with data from the same radio collars in this ecosystem, see Goodheart et al. (2022).

### 2.4 Criteria for Data Inclusion

**FIGURE 1:**
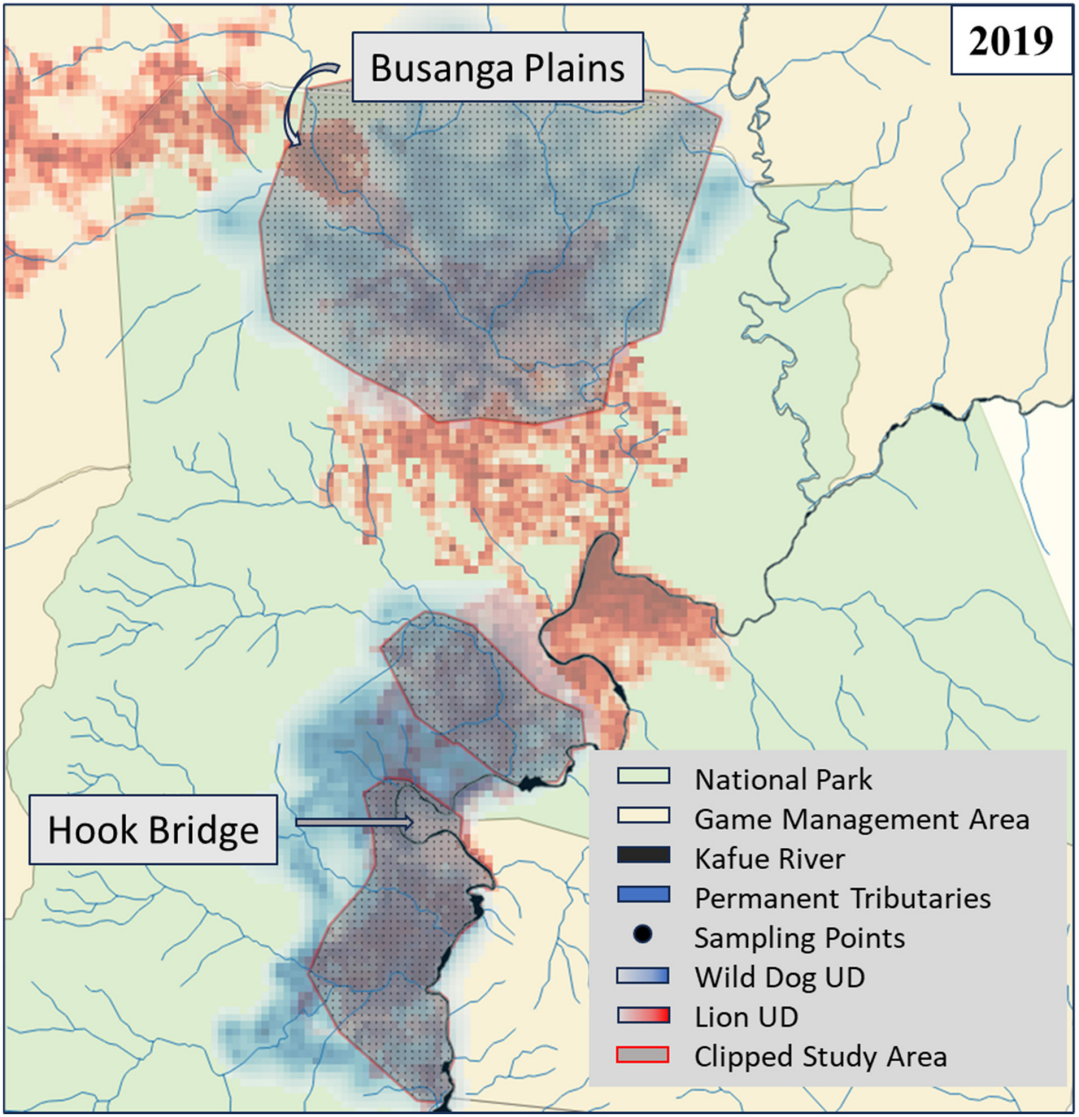
An example of a full year investigation showing overlaid utilization distribution of lions (red) and wild dogs (blue) for year 2019. Gray polygons outlined in red denote clipped study areas in which lions and wild dogs were well monitored (see: Criteria for inclusion) in 2019 only. Note that we included areas totally avoided by wild dogs and lions but with good monitoring from long‐term field studies and citizen science in those areas, so that local absence of wild dogs or lions was well documented (Busanga Plains & Hook Bridge). Sampled points along a stratified grid within the clipped areas from which UD values of both lions and wild dogs were extracted, are denoted in black.

For a valid test of the effect of lions on wild dog space‐use, it was necessary to restrict our study area to regions in which both lion and wild dog space‐use was well‐measured. Intensive monitoring of large carnivores is difficult, and uniform monitoring of large carnivores across space and time is not always possible. As in our prior work (Goodheart et al. 2022), we avoided analysis of data from areas in which incomplete monitoring of either species could be misinterpreted as a lack of use, by restricting analysis to areas with well‐monitored resident groups for both wild dogs and lions. We created 95% isopleths calculated from dBBMMs for each monitored pack and pride, exported them from R as polygons and overlaid them using QGIS 3.26 (www.QGIS.org) to delineate a study area for each year and season, in which both lion and wild dog space‐use was well described with location data covering ≥ 75% of the associated year or season, with no uncollared resident wild dog packs or lion prides utilizing the same area. Polygons were extended into well monitored areas beyond these boundaries only if apparent absence of either wild dogs or lions was well documented based on 4270 person days in the field from 2018 to 2022 (Figure 1) and intensive monitoring in these areas by citizen science and close collaboration with safari operators, managers, and other conservation organizations. For example, our long‐term monitoring program combined with our long‐standing citizen science partnership in the Busanga plains region of Kafue National Park has detected no resident wild dog packs and only occasional dispersing groups, but it is heavily utilized by lions and holds the largest prides of lions in the GKE and was therefore included in analysis (Figure 1). At no point during our study period did our intensive monitoring efforts and citizen science program detect unknown resident groups of lions or wild dogs within the polygons included in this analysis. Thus, the analysis excludes areas in which an apparent lack of use is an artifact of monitoring effort.

### 2.5 Data Extraction

Within each of the 15 sampling boundaries just described, we sampled points from a stratified grid of 1000 m in both directions (Marzluff et al. 2004; Dröge et al. 2017b) using the SP package in R (Pebesma et al. 2021). At each point, we extracted UD values for wild dogs and lions using the terra package (Hijmans et al. 2022). This procedure ensured balanced data from areas that were little‐used and areas that were heavily used. To account for spatial autocorrelation, we incorporated an (AR1) autoregression term for every sample, by including the UD value of the nearest neighbor as a variable in each model. Temporal autocorrelation is directly accounted for within the dBBMM (Kranstauber et al. 2012).

**FIGURE 2:**
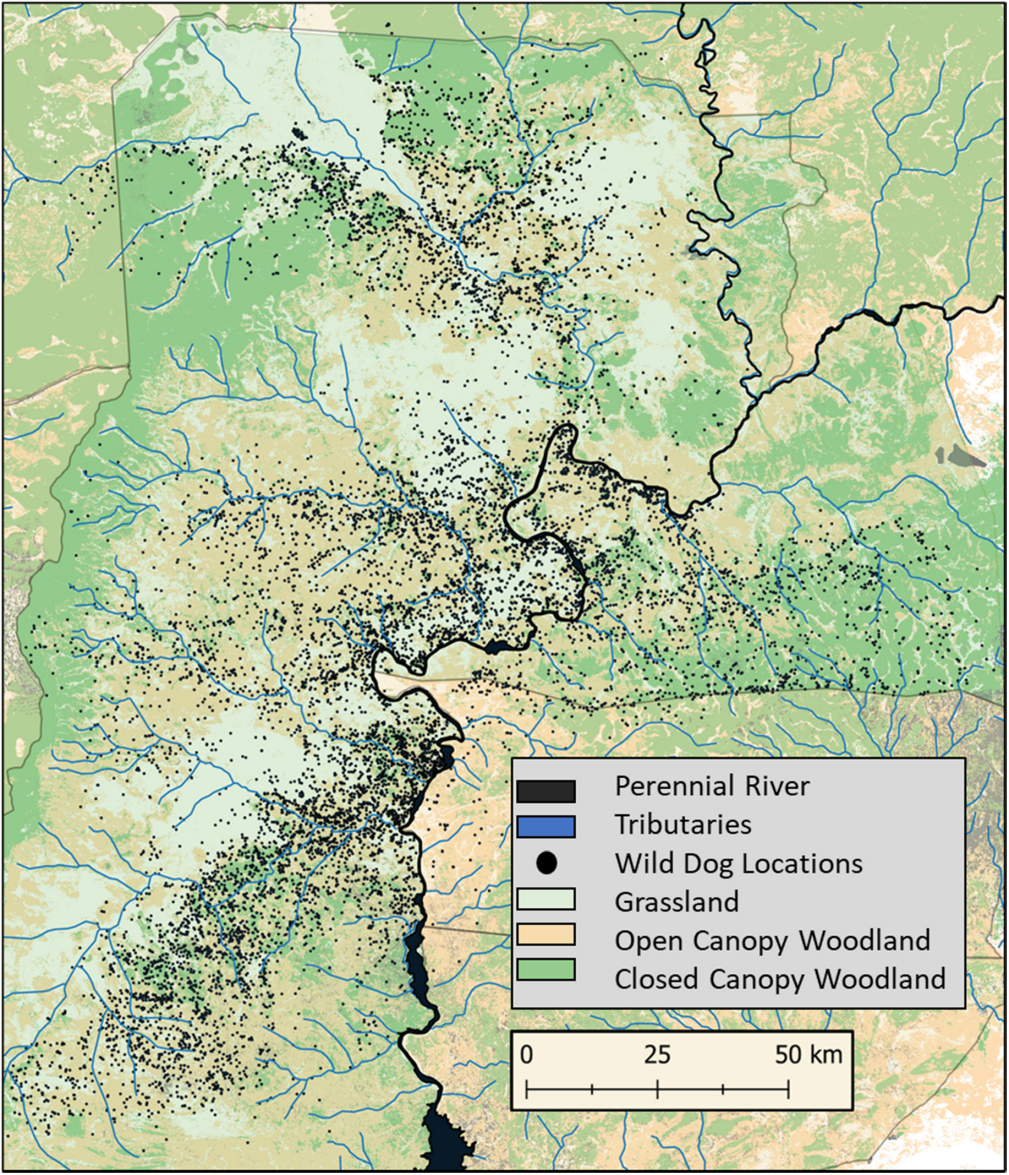
Map of study area showing the distribution of all wild dogs locations and vegetation types used in the analysis.

We tested for effects of biotic variables (vegetation class, edge density, and distance to water) that predict density and distribution of three dominant prey species for wild dogs in the GKE (Creel et al. 2018). Puku (*Kobus vardonii*) are associated with grasslands in close proximity to water, impala (*Aepyceros melampus*) are associated with open woodlands and heterogeneous habitats in close proximity to water, and common duiker (*Sylvicapra grimmia*) are associated with open and closed woodlands further from water sources (Rduch 2014; Matandiko 2016; Schuette et al. 2018; Vinks et al. 2020). Vegetation type was divided into four dominant classes (Fanshawe 2010) (Figure 2): closed canopy woodland (combretum thickets, riverine forests, and gallery miombo woodland), open canopy woodland (miombo, acacia, and termitaria woodland savannah), grassland (open plains, dambos, and floodplains), and human (farmland, cleared forests, or other uses). These were extracted from a raster layer with 30 m resolution created for the Kavango‐Zambezi Transfrontier Conservation Area from remote sensed data in 2016 and updated in 2020 (https://panda.maps.arcgis.com/home/item.html?id=b9459f0149794320b9cf7cc15935e858, accessed June 7, 2022). Vegetation was sampled within the same 1 km^2^ cells as wild dog UD values within the delineated sampling boundary. Percent‐cover of the raster cell was calculated for each landcover type, and the dominant landcover type was assigned to the cell for further analysis. Additionally, the Shannon Diversity Index was calculated to measure habitat heterogeneity for each cell, using the vegan package in R (Oksanen et al. 2007). Distances from centroids of the sampled raster cell to water were measured in two ways using the gDistance package in R (van Etten 2017): (1) Distance to perennial rivers: the Kafue River and its tributary the Lunga River, which most of the Northern GKE and our study area is centered upon. (2) Distance to any water: tributaries to the perennial rivers, or the perennial river itself if no tributary is closer. These tributaries are characterized by intermittent flow but hold water year‐round in pools along the stream bed, the entirety of which lie within the Kafue River watershed. In addition to including “human” land‐use among the vegetation types, we tested anthropogenic variables previously shown to affect wild dog movements and ungulate density in the GKE, which we hypothesized might also affect wild dog space‐use (Schuette et al. 2018; Vinks et al. 2020; Goodheart et al. 2022): (1) protection level (See *Study Area*: National Park, Game Management Area, Unprotected) and (2) distance to the nearest road.

### 2.6 Statistical Analysis

**FIGURE A1:**
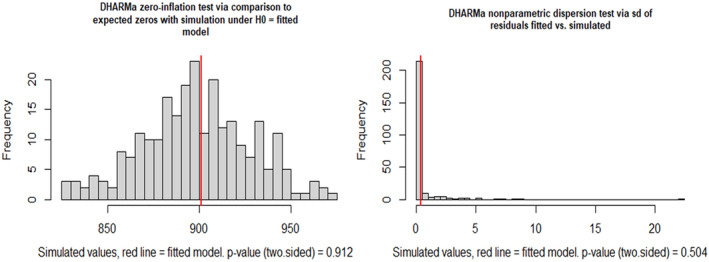
Goodness of fit test for our model of zero‐inflation (left) in patterns of space use showing the fitted zero‐inflated model did a good job of predicting zeros under simulation. A dispersion test (right) shows that dispersion under the fitted model is not significant. Plots above are for the zero‐inflated gamma model fit to our non‐weighted full‐year dataset. All of our models had similar results.

**FIGURE A2:**
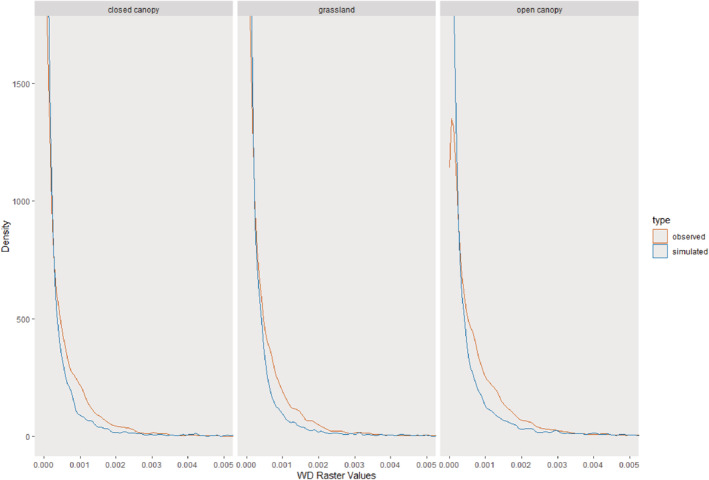
An assessment of goodness of fit for our full‐year zero‐inflated gamma model. Across habitats, the distribution of y‐hat values from the model (orange) closely matched the distribution of observed values (blue).

Using a hypothesis testing approach, we fit a generalized linear model with the set of predictors described above that we hypothesized could affect wild dog space‐use. As described, the model included an AR1 term to account for spatial autocorrelation between points. We combined all wet season data, all dry season data, and all full‐year data into three datasets and ran identically structured models on all three datasets to determine effects of the same set of predictors at seasonal and yearly timescales. For all models, the dependent variable was the wild dog UD value generated by dBBMMs. Our dependent variable contained a high frequency of zeros and was highly skewed, with a point mass approaching zero because we included areas in which lions and wild dogs were known to be absent based on our intensive monitoring efforts (see criteria for data inclusion). Therefore, we fit zero‐inflated GLMs with a gamma distribution using the glmmTMB package in R version 4.3.1 (Magnusson et al. 2017) to all three temporal datasets, plus an additional three models in which the lion‐space use variable was weighted by pride size. We tested for zero‐inflation and dispersion using the DHARMa package in R (Hartig 2021) and confirmed that our zero‐inflated gamma regression predicted zeros well (*p*‐values for all models were between 0.9 and 1) and residuals did not show significant overdispersion (Figure A1). Inferences were made from the count process of the model (i.e., testing effects on intensity of space‐use only in the places that were used). The null model for zero inflation had little influence on the count process and had no effects on the inferences, as demonstrated by fitting a GLM with a gamma distribution using the same glmTMB package (Magnusson et al. 2017) to the same dataset after removing all zeros (Table S1). We centered and scaled all continuous variables to directly compare the strength of effects and improve model convergence. We incorporated a random effect of year on the models' intercept, but found no changes in results or inferences, thus we did not include the random effect for ease of interpretation. We tested interactions between variables we believed could influence wild dog space‐use a priori: (1) Interaction between lion utilization and dominant habitat type, (2) lion utilization and distance to water, (3) A three‐way interaction between the previous three variables, however, AIC and log likelihood scores did not support inclusion of these effects in the model. We compared subsets of observed data defined by categorical predictors to simulated data from our model to further confirm goodness of fit (Figure A2). We tested multicollinearity among our continuous predictors and found that all generalized variance inflation factor values were < 2.

#### 2.6.1 Lions

**FIGURE A3:**
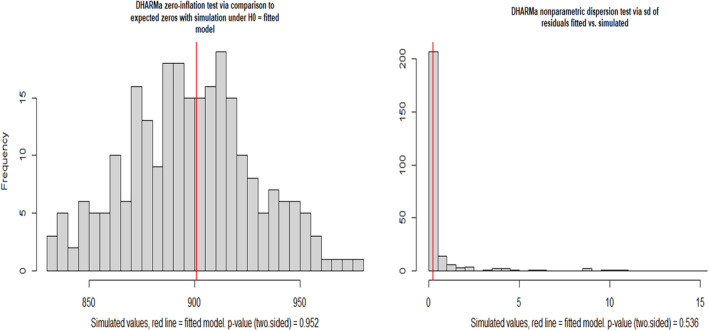
Lion analysis tests of zero‐inflation (left) showing the fitted zero‐inflated model does a good job of predicting zeros under simulation. A dispersion test (right) shows that dispersion under the fitted model is not significant. Plots above are for the zero‐inflated gamma model fit to our non‐weighted full‐year dataset. All six of our models had similar results as above.

**FIGURE A4:**
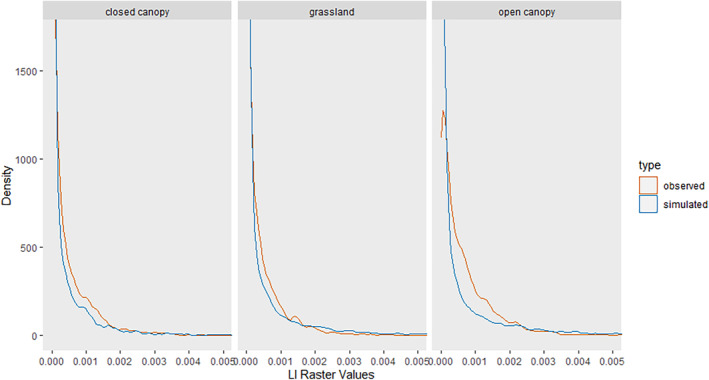
An assessment of goodness of fit for our full‐year zero‐inflated gamma model assessing lion space‐use. Across habitats, the distribution of y‐hat values from the model (orange) closely matched the distribution of observed values (blue).

Using identical methods to those for wild dogs, we fit models with the same set of predictors to determine these effects on lion space‐use. Prey depletion in the GKE has led to increased dietary overlap between wild dogs and lions (Creel et al. 2018) and so we would expect similar utilization of habitat as a result. Briefly, we sampled lion utilization values generated by dBBMMs within the same study area and combined these data for dry season, wet season, and full year investigations in the same manner as our wild dog analyses. Lion utilization distribution values were the dependent variable, and we tested for effects of the same set of environmental and anthropogenic predictors on these UD values, using identically structured models, including an (AR1) spatial autoregression term. We fit zero‐inflated GLMs with a gamma distribution, and centered and scaled all continuous predictor variables to directly compare strength of effects and improve convergence. To evaluate goodness of fit, we tested for zero‐inflation and over‐dispersion, and compared subsets of observed data defined by categorical variables to simulated data from our models (Figures A3 and A4).

## 3 Results

### 3.1 Effects of Lions on Wild Dog Space‐Use

**FIGURE 3:**
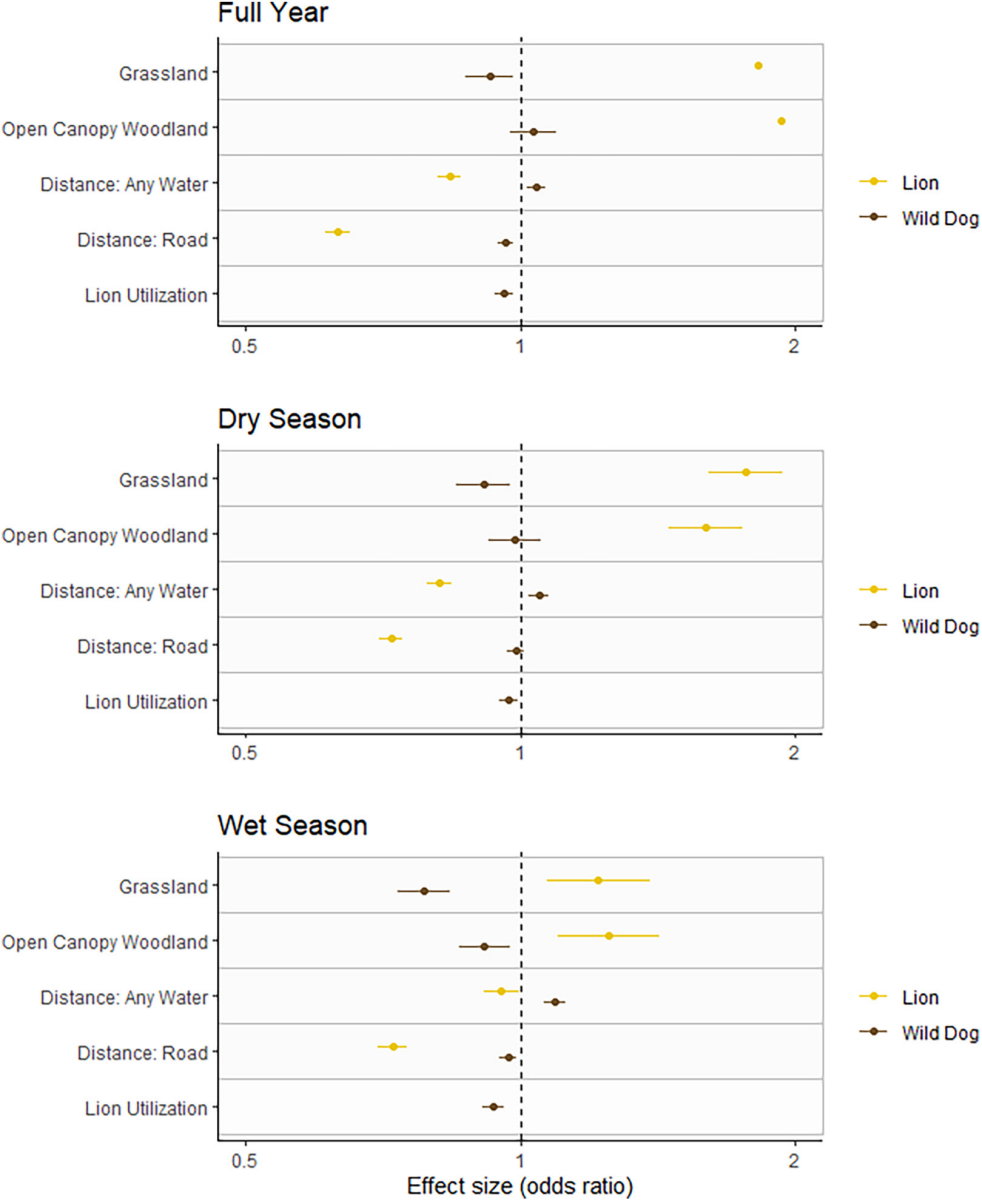
Effects from selected environmental, competitive, and anthropogenic variables on wild dog and lion space‐use in the GKE, across full years and seasons. The dashed line separates positive from negative parameter effects (note that positive effects for distance variables indicate utilization of areas further away from the variable). Wild dogs were negatively associated with areas more intensively utilized by lions at all timescales. Wild dogs also showed a strong negative association with grasslands, and utilized areas further from water sources, in contrast to lions.

Despite the low density of lions in the GKE, wild dog use was negatively associated with areas that were heavily used by lions over the course of a year (*b* = −0.041, SE = 0.011, *z* = −3.57, *p* < 0.001) (Table 1). Wild dogs showed a stronger negative association with lions during the wet season (*b* = −0.068, SE = 0.013, *z* = −5.13, *p* > 0.001) (Figure 3), than during the dry season (*b* = − 0.030, SE = 0.012, *z* = −2.37, *p* = 0.018). Weighting lion use by pride size produced only marginal changes in estimates of avoidance by wild dogs across years and seasons and did not substantially change estimated effects of other variables (Table S2).

**TABLE 1:** Analysis of effects on wild dog space‐use across years, dry season, wet seasons.

Wild dog space‐use models			
Full year			
Predictors	Estimates	CI	*p*
**Count model**			
(Intercept)	−8.62	−8.70 to −8.55	< 0.001
Autoregression term	1.57	1.52 to 1.62	< 0.001
**Lion utilization**	**−0.04**	−0.06 to −0.02	**< 0.001**
**Grassland**	**−0.08**	−0.14 to −0.02	**0.012**
Open canopy woodland	0.03	‐0.02 to 0.09	0.251
**Habitat heterogeneity**	**0.1**	0.08 to 0.13	**< 0.001**
**Distance: perennial river**	**−0.18**	−0.21 to −0.16	**< 0.001**
**Distance: any water**	**0.04**	0.02 to 0.06	**0.001**
**National park**	**0.67**	0.59 to 0.74	**< 0.001**
**No protection**	**−0.54**	−0.87 to −0.21	**0.001**
**Distance: road**	**−0.04**	−0.06 to −0.02	**< 0.001**
*Zero‐inflation model*			
(Intercept)	−2.66	−2.72 to −2.60	< 0.001
Observations	19,874		
*R* ^2^ marginal	0.684		

Dry season			
Predictors	Estimates	CI	*p*
**Count model**			
(Intercept)	−8.22	−8.29 to −8.15	< 0.001
Autoregression term	1.91	1.85 to 1.98	< 0.001
**Lion utilization**	**−0.03**	−0.05 to −0.01	**0.018**
**Grassland**	**−0.09**	−0.16 to −0.03	**0.007**
Open canopy woodland	−0.01	−0.08 to 0.05	0.675
**Habitat heterogeneity**	**0.11**	0.08 to 0.13	**< 0.001**
**Distance: perennial river**	**−0.15**	−0.18 to −0.12	**< 0.001**
**Distance: any water**	**0.05**	0.02 to 0.07	**< 0.001**
**National park**	**0.26**	0.20 to 0.33	**< 0.001**
**No protection**	**−0.86**	−1.41 to −0.31	**0.002**
Distance: road	−0.01	−0.03 to 0.01	0.322
*Zero‐inflation model*			
(Intercept)	−2.11	−2.15 to −2.06	< 0.001
Observations	21,140		
*R* ^2^ marginal	0.708		

Wet season			
Predictors	Estimates	CI	*p*
**Count model**			
(Intercept)	−8.23	−8.30 to −8.15	< 0.001
Autoregression term	1.32	1.28 to 1.37	< 0.001
**Lion utilization**	**−0.07**	−0.09 to −0.04	**< 0.001**
**Grassland**	**−0.24**	−0.31 to −0.18	**< 0.001**
**Open canopy woodland**	**−0.09**	−0.15 to −0.03	**0.006**
**Habitat heterogeneity**	**0.13**	0.10 to 0.15	**< 0.001**
**Distance: perennial river**	**−0.21**	−0.24 to −0.18	**< 0.001**
**Distance: any water**	**0.09**	0.06 to 0.11	**< 0.001**
**National park**	**0.56**	0.48 to 0.64	**< 0.001**
No protection	−0.02	−0.44 to 0.39	0.908
**Distance: road**	**−0.03**	−0.05 to −0.01	**0.007**
*Zero‐inflation model*			
(Intercept)	−2.35	−2.40 to −2.30	< 0.001
Observations	18,997		
*R* ^2^ marginal	0.656		

*Note:* Coefficient estimates are shown with associated confidence intervals (CI) and *p*‐values denoted in bold lettering when *p* < 0.05. Note negative associations with water, grassland habitats, and areas used heavily by lions across all temporal scales. “Observations” indicate the number of sampled points in the model.

### 3.2 Effects of Predictors of Prey Density on Wild Dog Space‐Use

Wild dog space‐use was negatively associated with habitats associated with puku (grasslands and close proximity to water) and partially those of impala (close proximity to water) the two most preferred prey species for wild dogs in the GKE. At all timescales, wild dogs were negatively associated with grasslands and water (Table 1 and Figure 3), however wild dog space‐use consistently decreased further from the Kafue River, on which the core of protection, herbivore densities, and our study areas are centered (Table 1). Wild dogs showed positive associations with habitat heterogeneity and woodlands across all timescales, except for a negative association with open‐canopy woodlands in relation to closed canopy woodlands during the wet season (*b* = −0.090, SE = 0.033, *z* = −2.75, *p* = 0.006) (Table 1).

### 3.3 Anthropogenic Effects on Wild Dog Space‐Use

Across all time‐scales, wild dogs used areas within the national park substantially more than GMAs and unprotected areas, and showed a negative association with areas lacking designated protection (outside the National Park and GMA boundaries) across years and during the dry season (Full Year: *b* = −0.541, SE = 0.167, *z* = −3.66, *p* < 0.001) (Dry Season: *b* = −0.861, SE = 0.281, *z* = −3.06, *p* = 0.002). Wild dog space‐use was positively associated with roads across years (*b* = −0.036, SE = 0.010, *z* = −3.66, *p* < 0.001) and the wet season (*b* = −0.031, SE = 0.011, *z* = −2.71, *p* = 0.007), but did not show as strong evidence of association during the dry season (*b* = −0.011, SE = 0.011, *z* = −0.99, *p* = 0.322).

### 3.4 Effects of Predictors of Prey Density on Lion Space‐Use

Predictors of prey density affected the space‐use of lions very differently than that of wild dogs (Figure 3). Lion space‐use substantially decreased further from water across years and seasons but did not show associations (positive or negative) with the Kafue river (Table 2 and Figure 3). Lions showed strong positive associations with habitat heterogeneity (full year: *b* = 0.053, SE = 0.017, *z* = 3.06, *p* = 0.002), grasslands (full year: *b* = 0.601, SE = 0.050, *z* = 11.73, *p* < 0.001), open canopy woodlands (full year: *b* = 0.660, SE = 0.048, *z* = 13.84, *p* < 0.001) and correspondingly negative associations with closed canopy woodlands across all time periods (Table 2 and Figure 3).

**TABLE 2:** Analysis of effects on lion space‐use across years, dry season, wet seasons.

Lion space‐use models			
Full year			
Predictors	Estimates	CI	*p*
**Count model**			
(Intercept)	−7.97	−8.10 to −7.85	< 0.001
Autoregression term	1.23	1.16 to 1.30	< 0.001
**Grassland**	**0.60**	0.50 to 0.70	**< 0.001**
**Open canopy woodland**	**0.66**	0.56 to 0.75	**< 0.001**
**Habitat heterogeneity**	**0.05**	0.02 to 0.09	**0.002**
Distance: perennial river	−0.03	−0.06 to 0.00	0.069
**Distance: any water**	**−0.18**	−0.21 to −0.15	**< 0.001**
**National park**	**0.51**	0.40 to 0.62	**< 0.001**
No protection	0.38	−0.17 to 0.93	0.172
**Distance: road**	**−0.46**	−0.49 to −0.43	**< 0.001**
*Zero‐inflation model*			
(Intercept)	−1.62	−1.66 to −1.58	< 0.001
Observations	19,874		
*R* ^2^ marginal	0.724		

Dry season			
Predictors	Estimates	CI	*p*
Count model			
(Intercept)	−7.77	−7.88 to −7.66	< 0.001
Autoregression term	1.3	1.23 to 1.36	< 0.001
**Grassland**	**0.57**	0.48 to 0.66	**< 0.001**
**Open canopy woodland**	**0.47**	0.38 to 0.56	**< 0.001**
**Habitat heterogeneity**	**0.1**	0.07 to 0.13	**< 0.001**
Distance: perennial river	−0.01	−0.04 to 0.02	0.35
**Distance: any water**	**−0.2**	−0.23 to −0.17	**< 0.001**
**National park**	**0.54**	0.45 to 0.63	**< 0.001**
No protection	−0.09	−0.68 to 0.50	0.761
**Distance: road**	**−0.33**	−0.36 to −0.30	**< 0.001**
*Zero‐inflation model*			
(Intercept)	−1.64	−1.68 to −1.61	< 0.001
Observations	21,140		
*R* ^2^ marginal	0.7		

Wet season			
Predictors	Estimates	CI	*p*
**Count model**			
(Intercept)	−6.97	−7.14 to −6.81	< 0.001
Autoregression term	1.3	1.21 to 1.38	< 0.001
**Grassland**	**0.2**	0.07 to 0.33	**0.003**
**Open canopy woodland**	**0.22**	0.10 to 0.35	**0.001**
**Habitat heterogeneity**	**0.1**	0.06 to 0.14	**< 0.001**
Distance: perennial river	−0.03	−0.07 to 0.01	0.123
**Distance: any water**	**−0.05**	−0.09 to −0.00	**0.038**
National park	0.09	−0.04 to 0.23	0.183
No protection	0	−0.81 to 0.81	0.998
**Distance: road**	**−0.32**	−0.36 to −0.28	**< 0.001**
*Zero‐inflation model*			
(Intercept)	−0.92	−0.95 to −0.89	< 0.001
Observations	18,997		
*R* ^2^ marginal	0.591		

*Note:* Coefficient estimates are shown with associated confidence intervals (CI) and *p*‐values denoted in bold lettering when *p* < 0.05. Note positive associations with water and habitats dominated by grassland and open‐canopy woodlands. “Observations” indicate the number of sampled points in the model.

## 4 Discussion

Subordinate competitors such as African wild dogs avoid dominant competitors to avoid intraguild predation and kleptoparasitism (Creel and Creel 1996, 2002; Vanak et al. 2013; Darnell et al. 2014; Jackson et al. 2014). Spatial avoidance is likely to have energetic costs because wild dogs must move more to avoid competitors (Goodheart et al. 2022), and because of competitive exclusion from prey‐rich areas (Mills and Gorman 1997; Vanak et al. 2013; Swanson et al. 2014; Dröge et al. 2017b). While these responses (and any associated fitness costs) have evolved to mitigate competition and predation, the ways that they are employed, and the degree to which they are effective, has not previously been tested in the heavily human‐impacted landscapes characterizing much of the remaining wild dog range. In the core of the GKE, prey densities are 4–42 times lower than expected, given the vegetation type and rainfall for the ecosystem (Vinks et al. 2020), lion densities are approximately three times lower than expected, and the loss of large herbivores like cape buffalo (*Syncerus caffer*) has led to greater diet overlap within the large carnivore guild (Creel et al. 2018). Despite the low density of lions and prey in the GKE, our results show that wild dogs still show strong spatial avoidance of lions and their preferred habitats across years and seasons, and consequently avoid habitats with higher densities of their preferred prey.

Wild dogs avoided habitats associated with high densities of both lions and key prey species, despite substantially reduced densities in the ecosystem. Wild dog utilization decreased closer to water at all time scales, whereas lion utilization substantially increased (Figure 3). Puku and impala are the two most common prey species for wild dogs in the GKE (Creel et al. 2018), and increase in density closer to water (Matandiko 2016; Schuette et al. 2018). Puku also favor grassland habitats over woodland habitats (Rduch 2014) and wild dogs showed an opposite preference (Figures 2 and 3). Of the three most selected prey species in the GKE by wild dogs, wild dogs showed habitat preferences that aligned only with common duiker, which are common in open and closed canopy woodlands and increase in density further from water (Matandiko 2016; Schuette et al. 2018; Vinks et al. 2020). Of these prey species, common duiker are the smallest and least selected by lions (Creel et al. 2018). For wild dogs, woodlands further from water likely serve as a refuge with little use by lions, where wild dogs can maintain access to small prey species such as duiker. Wild dogs' avoidance of areas with higher densities of larger prey in the GKE is similar to findings in ecosystems with higher densities of lions, such as Kruger National Park, where wild dogs avoided areas with high densities of impala due to high intensity of lion use in those areas (Mills and Gorman 1997), and in the Selous Game Reserve, where wild dogs hunted preferentially in areas with low use by lions, even though this reduced their rate of encounter with prey (Gallagher et al. 2017). Wild dogs showed a positive association with heterogeneous and woodland habitats, indicating wild dogs may feel safer accessing areas with larger prey such as impala in heterogeneous landscapes like open woodland, rather than open grasslands, which were consistently avoided by wild dogs. In other ecosystems with greater densities of prey and lions, wild dogs showed similar preference for heterogeneous woodland habitats (Jackson et al. 2014; Dröge et al. 2017b; Bouley et al. 2021; Davies et al. 2021): heterogeneity is likely important in facilitating wild dog access to resources in areas with higher densities of lions both between and within ecosystems (Creel 2001).

**FIGURE 4:**
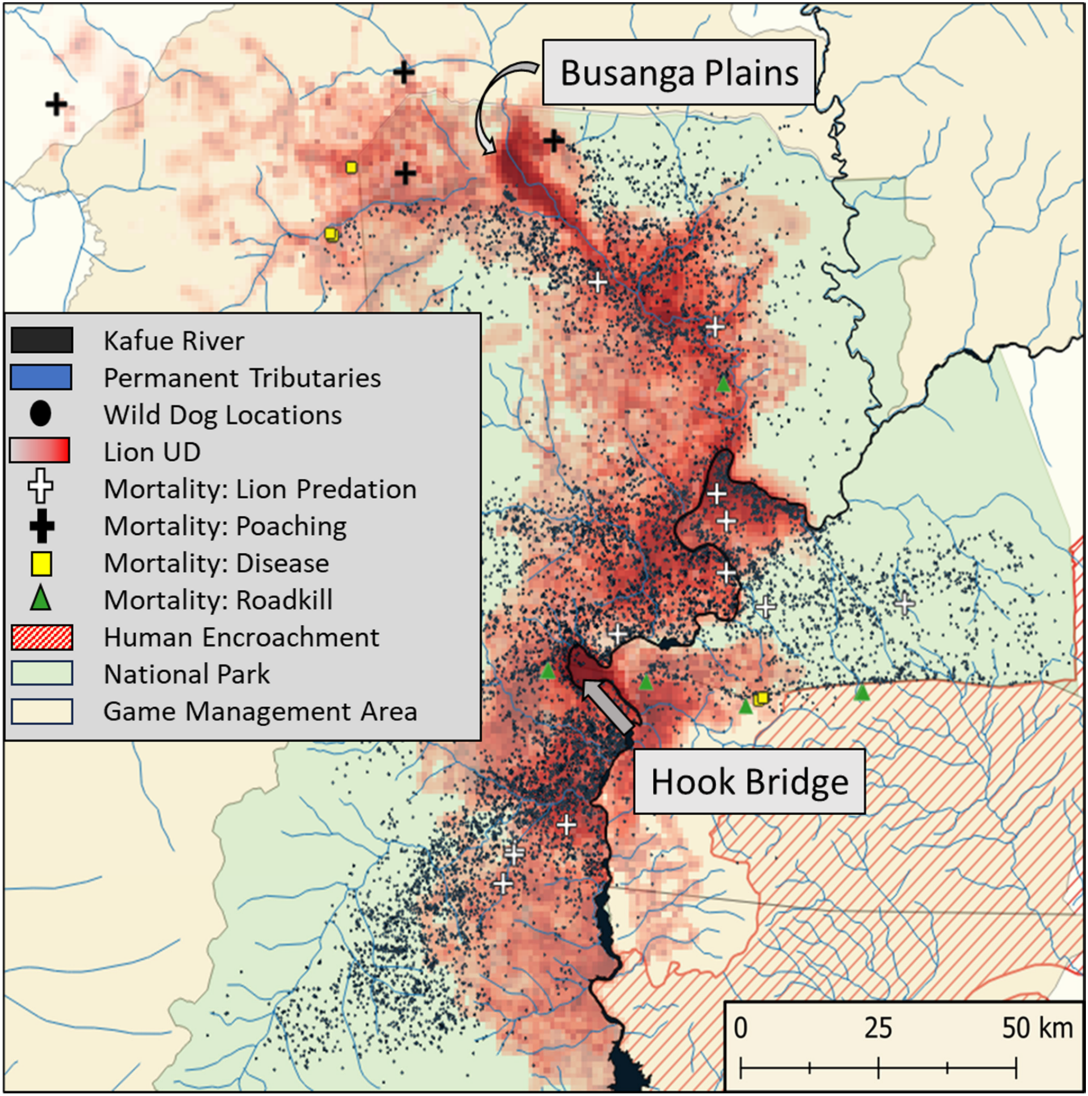
A map of all wild dog locations (points) plotted against combined lion utilization (red) across our entire study period. Visually, wild dogs showed avoidance of many areas that were heavily used by lions, most notably in the Busanga plains (upper left) and Hook Bridge area (Central), Both of which were well monitored, but never held a resident pack of wild dogs during the study period. Wild dogs also showed substantial avoidance of human encroachment in south‐east corner of the study area. All known wild dog mortality locations during the study period are plotted, with lion predation being the most common, followed by roadkill (which is likely to have the highest probability of detection), and disease (rabies).

Wild dog space‐use was negatively associated with areas that were most used by lions (Figures 1 and 3), but this basic pattern was not altered when we weighted lion space‐use by pride size (Table S1). In the GKE, our results indicate that wild dogs may avoid areas that are predictably used by lions, rather than assessing risk based on the number of lions in an area. Lions outweigh wild dogs 7 to 1 (Creel and Creel 1996), have a longer stride length, faster acceleration, and can kill a wild dog upon contact (personal observation, Creel and Creel 2002). Thus, it is intuitive that even one or two lions pose a serious risk of death to wild dogs and are worth avoiding if possible. Lion home‐ranges are influenced by the dispersion of prey resources (Mbizah et al. 2019) so that home‐range size increases as resources become more dispersed (Loveridge et al. 2009; Valeix, Loveridge, and Macdonald 2012). In the GKE, resident packs of wild dogs completely avoided areas where lions had the smallest and most predictable home‐ranges such as the Busanga plains and the Hook Bridge area, which both hold high densities of prey (Figures 1 and 4). Prey depletion may lead to larger lion home‐ranges, and thus less predictability in their use of specific locations, making avoidance of lions more difficult and energetically costly. In the GKE, the most known‐cause wild dog deaths were by lion predation (despite its low detectability). Lions accounted for 37% of 35 known‐cause deaths of wild dogs in the GKE, compared to 44% of 20 (Mills and Biggs 1993) in Kruger National Park and 27% of 11 in the Selous Game Reserve (Creel and Creel 1996), which have three times higher lion density (Goodheart et al. 2021). Lion predation events on wild dogs did not occur in areas most frequently used by lions (Figure 4), likely because wild dogs avoided them, and altered movements within these predictably risky areas (Goodheart et al. 2022).

Resident packs in our analysis overwhelmingly utilized the national park rather than the GMAs and unprotected areas. Our estimates of prey density in the GKE come from the core of the national park in the areas that are relatively well‐protected (Vinks et al. 2020). In many of the Kafue GMAs and surrounding unprotected areas, prey density is substantially lower relative to the park, due to increasing human impacts (Lindsey et al. 2014). Wild dogs do utilize GMAs and private conservancies with adequate protection, and these areas are important for population persistence, recovery, and connectivity; however, many of these packs were more susceptible to human impacts such as snares and disease. These threats combined with prey depletion likely resulted in the shorter pack tenures observed (ZCP unpublished data). While more study is needed to assess the dynamics across gradients of protection in this system, companion studies in Zambia's Luangwa Valley found source‐sink dynamics between national parks and GMAs (Creel et al. 2024) that are likely occurring in the GKE as well. Of 21 dispersal events with known outcomes for the monitored individuals, only two resulted in successful pack formation and breeding primarily outside the national park compared to 12 inside the park. Of the two packs that established outside the national park; one pack later succumbed to rabies shortly after breeding, and the alpha male was killed by a snare in the other (ZCP unpublished data). All other dispersers that left the national park either returned to their natal group or died, further supporting the idea of source‐sink dynamics across gradients of protection (Creel et al. 2024). We included a category for human altered landscapes in our habitat classification, but resident packs of wild dogs in the GKE avoided human altered landscapes so completely that we could not include this category in our analysis. Human encroachment in the form of slash and burn agriculture in the GKE has increased at alarming rates in recent years (Watson et al. 2015, ZCP, unpublished data). Wild dogs in the GKE utilized areas adjacent to encroachment (Figure 4); however, these areas are risky because they can have higher rates of snaring (Watson et al. 2013) and exposure to domestic dogs, increasing risk of disease transmission (Woodroffe and Donnelly 2011). Disease, primarily rabies transmitted by domestic dogs, is a serious concern for African wild dog conservation (Woodroffe and Ginsberg 1999; Prager et al. 2012; Woodroffe and Sillero‐Zubiri 2020) and accounted for 26% of known deaths in the GKE. These deaths occurred in areas that were near humans, or in fringe areas that lacked protection and had low densities of prey and lions, in which humans utilize domestic dogs (typically unvaccinated) to poach wildlife (Figure 4).

Bushmeat poaching has increased in many protected areas in Africa, reducing densities of prey and dominant carnivores such as lions. Wild dogs, which naturally occur at lower densities, decline in parallel with lions when prey density drops below a tipping point (Goodheart et al. 2021; Creel et al. 2023). We found that in an ecosystem with significantly reduced prey and lion density, wild dogs still avoid areas heavily used by lions, and their habitats. Wild dogs also avoided, and partially avoided habitat types selected by their two most selected prey species, even though densities of these prey species have been severely reduced. Prior research shows that foraging success decreases when wild dogs avoid lions and consequently hunt in habitats with lower prey density (Creel 2001), and this is driven mainly by a decrease in prey encounter rates (Creel and Creel 2002). Avoiding both lions and areas rich in medium sized ungulates likely have energetic costs that scale up to the population level. Wild dogs in the core of the GKE have survival rates comparable to ecosystems with higher densities of lions and prey, but pack size is substantially lower and packs cover massive home‐ranges (Goodheart et al. 2021). We have shown that low lion density in the GKE does not necessarily reduce the competitive top‐down effects of avoidance that lions impose on wild dogs. Reduced prey densities, coupled with prey‐base homogenization, may maintain or even increase competition with lions in the GKE, explaining the lack of competitive release by wild dogs shown by Goodheart et al. (2021) when lion densities decrease as a result of prey depletion. In the GKE and many other ecosystems affected by prey depletion, increasing the protection of prey species, specifically larger ungulates which have been severely reduced, should benefit the entire large carnivore guild and reduce niche overlap. Specifically targeting protection outside main photographic tourism zones with relatively high lion densities and reducing human encroachment on protected areas will likely have strong positive impacts on African wild dog populations. As prey depletion continues to affect ecosystems worldwide, the importance of understanding these impacts on carnivores and carnivore guild dynamics should be considered a key area of focus for conservation.

## Author Contributions

**Ben Goodheart:** conceptualization (lead), data curation (lead), formal analysis (lead), investigation (lead), methodology (equal), project administration (supporting), software (lead), validation (equal), visualization (lead), writing – original draft (lead), writing – review and editing (equal). **Scott Creel:** conceptualization (equal), formal analysis (supporting), funding acquisition (equal), methodology (equal), project administration (supporting), software (supporting), supervision (equal), validation (equal), writing – review and editing (lead). **Paul Schuette:** supervision (supporting), validation (supporting), writing – review and editing (supporting). **Egil Droge:** supervision (supporting), validation (supporting), writing – review and editing (supporting). **Justine A. Becker:** supervision (supporting), validation (supporting), writing – review and editing (supporting). **Kambwiri Banda:** investigation (supporting), project administration (supporting). **Anna Kusler:** investigation (supporting), project administration (supporting), writing – review and editing (supporting). **Stephi Matsushima:** investigation (supporting), project administration (supporting). **Kachama Banda:** investigation (supporting), project administration (supporting). **Ruth Kabwe:** investigation (supporting). **Will Donald:** investigation (supporting), writing – review and editing (supporting). **Johnathan Reyes de Merkle:** resources (supporting), writing – review and editing (supporting). **Adrian Kaluka:** project administration (supporting), resources (supporting). **Clive Chifunte:** investigation (supporting). **Matthew S. Becker:** funding acquisition (equal), project administration (lead), resources (lead), supervision (equal), writing – review and editing (equal).

## Conflicts of Interest

The authors declare no conflicts of interest.

## Supporting information


**Table S1.** Analysis of effects on wild dog space‐use across years, dry season, wet seasons after removing zeros from the response variable (wild dog UD values). Coefficient estimates are shown with associated confidence intervals (CI) and *p*‐values denoted in bold lettering when *p* < 0.05. Note the almost identical coefficient estimates and *p*‐values as the zero‐inflated models. “Observations” indicate the number of sampled points in the model.
**Table S2.** Effects on wild dog space‐use when lion utilization distributions are weighted by pride size. Coefficient estimates with confidence intervals (CI) and associated *p*‐values over a period of 1‐year, dry season, and wet season. Bold lettering denotes *p* < 0.05.

## Data Availability

Data is available at https://datadryad.org/stash/share/f8bxlAokXPzjMYwe1QSEutRhNexvG_MaJbM1Kc‐qU_0.
