# Supplementary material for: Spatial Risk Effects From Lions Compound Impacts of Prey Depletion on African Wild Dogs
Source: Ecol Evol. 2024 Oct 18;14(10):e70401. doi: 10.1002/ece3.70401 (PMC11489504; doi:10.1002/ece3.70401)
Supplement: Supplementary file 1 — Table S1. Analysis of effects on wild dog space‐use across years, dry season, wet seasons after removing zeros from the response variable (wild dog UD values). Coefficient estimates are shown with associated confidence intervals (CI) and p‐values denoted in bold lettering when p < 0.05. Note the almost identical coefficient estimates and p‐values as the zero‐inflated models. “Observations” indicate the number of sampled points in the model. Table S2. Effects on wild dog space‐use when lion utilization distributions are weighted by pride size. Coefficient estimates with confidence intervals (CI) and associated p‐values over a period of 1‐year, dry season, and wet season. Bold lettering denotes p < 0.05. [file ECE3-14-e70401-s001.pdf]

Wild dog space-use models (without zeros)

Full Year

Predictors	*Estimates*	*CI*	*p*
(Intercept)	-8.55	-8.62 – -8.47	<0.001
Autoregression term	1.61	1.56 – 1.66	<0.001
**Lion utilization**	**-0.04**	-0.06 – -0.02	**<0.001**
**Grassland**	**-0.08**	-0.14 – -0.02	**0.012**
Open canopy woodland	0.03	-0.02 – 0.09	0.251
**Habitat heterogeneity**	**0.1**	0.08 – 0.12	**<0.001**
**Distance: perennial river**	**-0.16**	-0.19 – -0.14	**<0.001**
**Distance: any water**	**0.04**	0.02 – 0.06	**0.001**
**National park**	**0.67**	0.59 – 0.74	**<0.001**
**No protection**	**-0.54**	-0.87 – -0.21	**0.001**
**Distance: road**	**-0.04**	-0.06 – -0.02	**<0.001**
Observations	18576		
R^2 marginal	0.704		

Dry Season

Predictors	*Estimates*	*CI*	*p*
(Intercept)	-8.12	-8.19 – -8.05	<0.001
Autoregression term	2.01	1.94 – 2.09	<0.001
**Lion utilization**	**-0.03**	-0.05 – -0.00	**0.018**
**Grassland**	**-0.09**	-0.16 – -0.03	**0.007**
Open canopy woodland	-0.01	-0.08 – 0.05	0.675
**Habitat heterogeneity**	**0.11**	0.08 – 0.13	**<0.001**
**Distance: perennial river**	**-0.14**	-0.16 – -0.11	**<0.001**
**Distance: any water**	**0.05**	0.02 – 0.07	**<0.001**
**National park**	**0.26**	0.20 – 0.33	**<0.001**
**No Protection**	**-0.86**	-1.41 – -0.31	**0.002**
Distance: road	-0.01	-0.03 – 0.01	0.322
Observations	18846		
R^2 marginal	0.745		

Wet Season

Predictors	*Estimates*	*CI*	*p*
(Intercept)	-8.13	-8.20 – -8.05	<0.001
Autoregression term	1.37	1.32 – 1.41	<0.001
**Lion Utilization**	**-0.06**	-0.09 – -0.04	**<0.001**
**Grassland**	**-0.24**	-0.31 – -0.18	**<0.001**
**Open canopy woodland**	**-0.09**	-0.16 – -0.03	**0.006**
**Habitat heterogeneity**	**0.13**	0.10 – 0.15	**<0.001**
**Distance: perennial river**	**-0.19**	-0.22 – -0.16	**<0.001**
**Distance: any water**	**0.09**	0.06 – 0.12	**<0.001**
**National Park**	**0.56**	0.48 – 0.64	**<0.001**
No Protection	-0.02	-0.44 – 0.39	0.907
**Distance: road**	**-0.03**	-0.05 – -0.01	**0.007**
Observations	17348		
R^2 marginal	0.686		

Table 1. Analysis of effects on wild dog space-use across years, dry season, wet seasons after removing zeros from the response variable (wild dog UD values). Coefficient estimates are shown with associated confidence intervals (CI) and p-values denoted in bold lettering when $p < 0.05$. Note the almost identical coefficient estimates and p-values as the zero-inflated models. "Observations" indicate the number of sampled points.

Wild dog space-use models (weighted by pride size)

Full Year

Predictors	*Estimates*	*CI*	*p*
**Count Model**			
(Intercept)	-8.63	-8.70 – -8.55	<0.001
Autoregression term	1.57	1.53 – 1.62	<0.001
**Lion utilization (weighted)**	**-0.03**	-0.05 – -0.00	**0.024**
**Grassland**	**-0.08**	-0.14 – -0.02	**0.007**
Open canopy woodland	0.03	-0.03 – 0.09	0.275
**Habitat heterogeneity**	**0.1**	0.08 – 0.12	**<0.001**
**Distance: perennial river**	**-0.18**	-0.21 – -0.15	**<0.001**
**Distance: any water**	**0.04**	0.02 – 0.07	**<0.001**
**National Park**	**0.67**	0.60 – 0.74	**<0.001**
**No Protection**	**-0.55**	-0.88 – -0.22	**0.001**
**Distance: road**	**-0.03**	-0.05 – -0.01	**<0.001**
Zero-Inflation Model			
(Intercept)	-2.66	-2.72 – -2.60	<0.001
Observations	19874		
R^2 marginal	0.683		

Dry Season

Predictors	*Estimates*	*CI*	*p*
**Count Model**			
(Intercept)	-8.22	-8.29 – -8.15	<0.001
Autoregression term	1.92	1.85 – 1.98	<0.001
**Lion utilization (weighted)**	**-0.04**	-0.06 – -0.01	**0.003**
**Grassland**	**-0.09**	-0.16 – -0.02	**0.008**
Open canopy woodland	-0.01	-0.08 – 0.05	0.704
**Habitat heterogeneity**	**0.11**	0.08 – 0.13	**<0.001**
**Distance: perennial river**	**-0.15**	-0.18 – -0.12	**<0.001**
**Distance: any water**	**0.05**	0.02 – 0.07	**<0.001**
**National Park**	**0.27**	0.20 – 0.34	**<0.001**
**No Protection**	**-0.88**	-1.42 – -0.33	**0.002**
Distance: road	-0.01	-0.03 – 0.01	0.334
Zero-Inflation Model			
(Intercept)	-2.11	-2.15 – -2.06	<0.001
Observations	21140		
R^2 marginal	0.708		

Wet Season

Predictors	*Estimates*	*CI*	*p*
**Count Model**			
(Intercept)	-8.23	-8.31 – -8.15	<0.001
Autoregression term	1.32	1.28 – 1.37	<0.001
**Lion utilization (weighted)**	**-0.08**	-0.11 – -0.06	**<0.001**
**Grassland**	**-0.24**	-0.31 – -0.17	**<0.001**
**Open canopy woodland**	**-0.09**	-0.16 – -0.03	**0.005**
**Habitat heterogeneity**	**0.13**	0.10 – 0.15	**<0.001**
**Distance: perennial river**	**-0.22**	-0.25 – -0.19	**<0.001**
**Distance: any water**	**0.09**	0.06 – 0.12	**<0.001**
**National Park**	**0.56**	0.48 – 0.64	**<0.001**
No Protection	-0.03	-0.44 – 0.39	0.90
**Distance: road**	**-0.03**	-0.05 – -0.01	**0.007**
Zero-Inflation Model			
(Intercept)	-2.35	-2.40 – -2.30	<0.001
Observations	18997		
R^2 marginal	0.657		

Table 2. Effects on wild dog space-use when lion utilization distributions are weighted by pride size. Coefficient estimates with confidence intervals (CI) and associated p-values over a period of one-year, dry season, and wet season. Bold lettering denotes $p < 0.05$.
